# Supplementary material for: Characteristics of Pica Behavior among Mothers around Lake Victoria, Kenya: A Cross-Sectional Study
Source: Int J Environ Res Public Health. 2019 Jul 14;16(14):2510. doi: 10.3390/ijerph16142510 (PMC6679355; doi:10.3390/ijerph16142510)
Supplement: Supplementary file 1 [file ijerph-16-02510-s001.zip › Supplementary File 1.docx]

Supplementary File 1. 24-hour Recall Survey – Mother

| Survey #_________________  Date of Interview_:_ //  Day Month Year | Participant ID:   Interviewer Initials:  |
| --- | --- |

| ***This form is to be completed for the mother.*** | | | | |
| --- | --- | --- | --- | --- |
| I would next like to ask you some questions about the foods you ate yesterday. Starting yesterday, I’m going to ask you about what, where, when, and how much you ate. Please tell me about foods you ate at home and away from home. Please explain exactly what you actually ate; there are no ‘good’ or ‘bad’ foods. | | | | |
| G.1 First Meal | | | | |
| G.1.1 | At what time did you eat this meal? | ^:^ | | |
| G.1.2 | a. What foods did you consume? | b. How much did you consume? | | |
|  | *Enter food and individual foods in brackets. For example sukuma wiki (tomatoes, onion), porridge (cassava, millet, sugar), tea (milk, sugar), omena soup (tomatoes, onion, oil).* | *Include amounts for all foods. Show respondents the items to reference amounts: cup, bowl, serving spoon, etc.*  *Questions to ask: How much did you use? How many did you eat? What size were the foods?* | | |
| G.1.2.1 | _________________________ | Unit _1_ ⭘ cup  _2_ ⭘ bowl  _3_ ⭘ table spoon  _4_ ⭘ serving spoon  _5_ ⭘ ‘ugali’ piece  _6_ ⭘ piece (for maize, fruit, etc)  _7_ ⭘ other, specify______________ | | Number of unit  __________________ |
| G.1.2.2 | _________________________ | Unit _1_ ⭘ cup  _2_ ⭘ bowl  _3_ ⭘ table spoon  _4_ ⭘ serving spoon  _5_ ⭘ ‘ugali’ piece  _6_ ⭘ piece (for maize, fruit, etc)  _7_ ⭘ other, specify______________ | | Number of unit  __________________ |
| G.1.2.3 | _________________________ | Unit _1_ ⭘ cup  _2_ ⭘ bowl  _3_ ⭘ table spoon  _4_ ⭘ serving spoon  _5_ ⭘ ‘ugali’ piece  _6_ ⭘ piece (for maize, fruit, etc)  _7_ ⭘ other, specify______________ | | Number of unit  __________________ |
| G.1.2.4 | _________________________ | Unit _1_ ⭘ cup  _2_ ⭘ bowl  _3_ ⭘ table spoon  _4_ ⭘ serving spoon  _5_ ⭘ ‘ugali’ piece  _6_ ⭘ piece (for maize, fruit, etc)  _7_ ⭘ other, specify______________ | Number of unit  __________________ | |
| G.1.2.5 | __________________________ | Unit _1_ ⭘ cup  _2_ ⭘ bowl  _3_ ⭘ table spoon  _4_ ⭘ serving spoon  _5_ ⭘ ‘ugali’ piece  _6_ ⭘ piece (for maize, fruit, etc)  _7_ ⭘ other, specify______________ | Number of unit  __________________ | |
| G.1.2.6 | __________________________ | Unit _1_ ⭘ cup  _2_ ⭘ bowl  _3_ ⭘ table spoon  _4_ ⭘ serving spoon  _5_ ⭘ ‘ugali’ piece  _6_ ⭘ piece (for maize, fruit, etc)  _7_ ⭘ other, specify______________ | | Number of unit  __________________ |
| G.2 Second Meal | | | | |
| G.2.1 | At what time did you eat this meal? | ^:^ | | |
| G.2.2 | a. What foods did you consume? | b. How much did you consume? | | |
| G.2.2.1 | __________________________ | Unit _1_ ⭘ cup  _2_ ⭘ bowl  _3_ ⭘ table spoon  _4_ ⭘ serving spoon  _5_ ⭘ ‘ugali’ piece  _6_ ⭘ piece (for maize, fruit, etc)  _7_ ⭘ other, specify______________ | | Number of unit  __________________ |
| G.2.2.2 | __________________________ | Unit _1_ ⭘ cup  _2_ ⭘ bowl  _3_ ⭘ table spoon  _4_ ⭘ serving spoon  _5_ ⭘ ‘ugali’ piece  _6_ ⭘ piece (for maize, fruit, etc)  _7_ ⭘ other, specify______________ | | Number of unit  __________________ |
| G.2.2.3 | __________________________ | Unit _1_ ⭘ cup  _2_ ⭘ bowl  _3_ ⭘ table spoon  _4_ ⭘ serving spoon  _5_ ⭘ ‘ugali’ piece  _6_ ⭘ piece (for maize, fruit, etc)  _7_ ⭘ other, specify______________ | | Number of unit  __________________ |
| G.2.2.4 | __________________________ | Unit _1_ ⭘ cup  _2_ ⭘ bowl  _3_ ⭘ table spoon  _4_ ⭘ serving spoon  _5_ ⭘ ‘ugali’ piece  _6_ ⭘ piece (for maize, fruit, etc)  _7_ ⭘ other, specify______________ | Number of unit  __________________ | |
| G.2.2.5 | __________________________ | Unit _1_ ⭘ cup  _2_ ⭘ bowl  _3_ ⭘ table spoon  _4_ ⭘ serving spoon  _5_ ⭘ ‘ugali’ piece  _6_ ⭘ piece (for maize, fruit, etc)  _7_ ⭘ other, specify______________ | Number of unit  __________________ | |
| G.2.2.6 | __________________________ | Unit _1_ ⭘ cup  _2_ ⭘ bowl  _3_ ⭘ table spoon  _4_ ⭘ serving spoon  _5_ ⭘ ‘ugali’ piece  _6_ ⭘ piece (for maize, fruit, etc)  _7_ ⭘ other, specify______________ | Number of unit  __________________ | |
| G.3 Third Meal | | | | |
| G.3.1 | At what time did you eat this meal? | ^:^ | | |
| G.3.2 | a. What foods did you consume? | b. How much did you consume? | | |
| G.3.2.1 | __________________________ | Unit _1_ ⭘ cup  _2_ ⭘ bowl  _3_ ⭘ table spoon  _4_ ⭘ serving spoon  _5_ ⭘ ‘ugali’ piece  _6_ ⭘ piece (for maize, fruit, etc)  _7_ ⭘ other, specify______________ | | Number of unit  __________________ |
| G.3.2.2 | __________________________ | Unit _1_ ⭘ cup  _2_ ⭘ bowl  _3_ ⭘ table spoon  _4_ ⭘ serving spoon  _5_ ⭘ ‘ugali’ piece  _6_ ⭘ piece (for maize, fruit, etc)  _7_ ⭘ other, specify______________ | | Number of unit  __________________ |
| G.3.2.3 | __________________________ | Unit _1_ ⭘ cup  _2_ ⭘ bowl  _3_ ⭘ table spoon  _4_ ⭘ serving spoon  _5_ ⭘ ‘ugali’ piece  _6_ ⭘ piece (for maize, fruit, etc)  _7_ ⭘ other, specify______________ | | Number of unit  __________________ |
| G.3.2.4 | __________________________ | Unit _1_ ⭘ cup  _2_ ⭘ bowl  _3_ ⭘ table spoon  _4_ ⭘ serving spoon  _5_ ⭘ ‘ugali’ piece  _6_ ⭘ piece (for maize, fruit, etc)  _7_ ⭘ other, specify______________ | | Number of unit  __________________ |
| G.3.2.5 | __________________________ | Unit _1_ ⭘ cup  _2_ ⭘ bowl  _3_ ⭘ table spoon  _4_ ⭘ serving spoon  _5_ ⭘ ‘ugali’ piece  _6_ ⭘ piece (for maize, fruit, etc)  _7_ ⭘ other, specify______________ | | Number of unit  __________________ |
| G.3.2.6 | __________________________ | Unit _1_ ⭘ cup  _2_ ⭘ bowl  _3_ ⭘ table spoon  _4_ ⭘ serving spoon  _5_ ⭘ ‘ugali’ piece  _6_ ⭘ piece (for maize, fruit, etc)  _7_ ⭘ other, specify______________ | | Number of unit  __________________ |
| G.4 Other Meals or Snacks | | | | |
| G.4.1 | a. What foods did you consume? | b. How much did you consume? | | |
| G.4.1.1 | __________________________ | Unit _1_ ⭘ cup  _2_ ⭘ bowl  _3_ ⭘ table spoon  _4_ ⭘ serving spoon  _5_ ⭘ ‘ugali’ piece  _6_ ⭘ piece (for maize, fruit, etc)  _7_ ⭘ other, specify______________ | | Number of unit  __________________ |
| G.4.1.2 | __________________________ | Unit _1_ ⭘ cup  _2_ ⭘ bowl  _3_ ⭘ table spoon  _4_ ⭘ serving spoon  _5_ ⭘ ‘ugali’ piece  _6_ ⭘ piece (for maize, fruit, etc)  _7_ ⭘ other, specify______________ | | Number of unit  __________________ |
| G.4.1.3 | __________________________ | Unit _1_ ⭘ cup  _2_ ⭘ bowl  _3_ ⭘ table spoon  _4_ ⭘ serving spoon  _5_ ⭘ ‘ugali’ piece  _6_ ⭘ piece (for maize, fruit, etc)  _7_ ⭘ other, specify______________ | | Number of unit  __________________ |
| G.4.1.4 | __________________________ | Unit _1_ ⭘ cup  _2_ ⭘ bowl  _3_ ⭘ table spoon  _4_ ⭘ serving spoon  _5_ ⭘ ‘ugali’ piece  _6_ ⭘ piece (for maize, fruit, etc)  _7_ ⭘ other, specify______________ | | Number of unit  __________________ |
| G.4.1.5 | __________________________ | Unit _1_ ⭘ cup  _2_ ⭘ bowl  _3_ ⭘ table spoon  _4_ ⭘ serving spoon  _5_ ⭘ ‘ugali’ piece  _6_ ⭘ piece (for maize, fruit, etc)  _7_ ⭘ other, specify______________ | | Number of unit  __________________ |
| G.4.1.6 | __________________________ | Unit _1_ ⭘ cup  _2_ ⭘ bowl  _3_ ⭘ table spoon  _4_ ⭘ serving spoon  _5_ ⭘ ‘ugali’ piece  _6_ ⭘ piece (for maize, fruit, etc)  _7_ ⭘ other, specify______________ | | Number of unit  __________________ |

| **G.5 Special Events** | | |
| --- | --- | --- |
| G.5.1 | Was the amount of food you ate yesterday unusual? | _1_ ⭘ Yes  _2_ ⭘ No 🡪 Skip to G.5.3 |
| G.5.2 | How was the amount of food you ate unusual? | _1_ ⭘ More than usual  _2_ ⭘ Less than usual  _3_ ⭘ Other, specify_________________________ |
| G.5.3 | Were you ill yesterday? | _1_ ⭘ Yes  _2_ ⭘ No 🡪 Skip to G.5.6 |
| G.5.4 | Did the illness affect your appetite? | _1_ ⭘ Yes  _2_ ⭘ No 🡪 Skip to G.5.6 |
| G.5.5 | How did the illness affect your appetite? | _1_ ⭘ Increased appetite  _2_ ⭘ Decreased appetite  _3_ ⭘ No effect |
| G.5.6 | Did you consume any tablets/ herbs/medicine yesterday? | _1_ ⭘ Yes  _2_ ⭘ No 🡪 SKIP to G.5.9 |
| G.5.7 | Which tablets/herbs/medicine did you consume? (Check all that apply) | _1_ ⭘ Multi-vitamin  _2_ ⭘ Iron  _3_ ⭘ Anti-malaria  _4_ ⭘ Anti-Retroviral Medication  _5_ ⭘ Dewormer  _6_ ⭘ Herbs  _7_ ⭘ Other, specify_________________________ |
| G.5.8 | How did the tablets/herbs/medicine affect your appetite? | _1_ ⭘ Increased appetite  _2_ ⭘ Decreased appetite  _3_ ⭘ No effect |
| G.5.9 | Did you take any deworming medications in the last 3 months?  *Be ne imuonyo yadh njofni moro amora ei dweche 3 mokalo?*  *Medicine for hookworm, ringworm, etc, examples are Albendazole or Mebendazole; does NOT include medicine for bilharzia/shistosomiasis* | _1_ ⭘ Yes  _2_ ⭘ No |

| **G.6 Non-foods/Uncooked Foods** Did you eat any of the following items yesterday? | | |
| --- | --- | --- |
| G.6.1 | Ash | _1_ ⭘ Yes _2_ ⭘ No |
| G.6.2 | Charcoal | _1_ ⭘ Yes _2_ ⭘ No |
| G.6.3 | Odowa | _1_ ⭘ Yes _2_ ⭘ No |
| G.6.4 | Soil, other | _1_ ⭘ Yes _2_ ⭘ No |
| G.6.5 | Uncooked food (cassava, rice, etc), specify______________ | _1_ ⭘ Yes _2_ ⭘ No |
| G.6.6 | Other non-food, specify________ | _1_ ⭘ Yes _2_ ⭘ No |
| G.7 | **Notes:** | |
