# Supplementary material for: Characteristics of Pica Behavior among Mothers around Lake Victoria, Kenya: A Cross-Sectional Study
Source: Int J Environ Res Public Health. 2019 Jul 14;16(14):2510. doi: 10.3390/ijerph16142510 (PMC6679355; doi:10.3390/ijerph16142510)
Supplement: Supplementary file 1 [file ijerph-16-02510-s001.zip › Supplementary File 2 v2.docx]

**Supplementary File 2.** Correlates of any pica, geophagy/charcoal/ash, and amylophagy including Interviewer Fixed Effects, Mfangano Island, Kenya*.

|  | **Any Pica**  **(n = 299)** |  | | **Geophagy/Charcoal/Ash**  **(n = 288)**§ | |  | **Amylophagy**  **(n = 299)** |
| --- | --- | --- | --- | --- | --- | --- | --- |
|  | OR (95% CI) | |  | | OR (95% CI) |  | OR (95% CI) |
| Currently Pregnant | 2.29 (0.68-7.68) | |  | | 1.11 (0.27-4.49) |  | 4.34 (1.18-15.91) |
| Currently Breastfeeding | 0.57 (0.29-1.11) | |  | | 0.40 (0.18-0.88) |  | 1.19 (0.50-2.81) |
| Maternal Morbidity Score | 0.78 (0.57-1.08) | |  | | 0.92 (0.61-1.38) |  | 0.75 (0.50-1.11) |
| Household Food Insecurity Score | 1.05 (0.99-1.13) | |  | | 1.04 (0.96-1.13) |  | 1.02 (0.94-1.11) |
| Region |  | |  | |  |  |  |
| 1 | Referent | |  | | Referent |  | Referent |
| 2 | 1.03 (0.25-4.23) | |  | | 1.69 (0.34-8.44) |  | 0.66 (0.10-4.18) |
| 3 | 1.55 (0.67-3.60) | |  | | 1.35 (0.49-3.69) |  | 1.23 (0.41-3.74) |
| 4 | 1.26 (0.47-3.36) | |  | | 0.60 (0.16-2.35) |  | 2.07 (0.64-6.66) |
| 5 | 1.03 (0.40-2.66) | |  | | 0.76 (0.23-2.50) |  | 1.33 (0.42-4.29) |
| 6 | 3.79 (1.41-10.20) | |  | | 4.24 (1.37-13.14) |  | 2.08 (0.60-7.22) |

* Separate multivariable models were run for any pica, geophagy/charcoal/ash, and amylophagy. All models controlled for maternal age, education, household assets, number of people in the household, and interviewer fixed effects.

§ For the geophagy, charcoal, or ash consumption model, only 288 observations were used because one interviewer had 11 observations which did not have any variability. All 11 households reported no geophagy, charcoal, or ash consumption; therefore, these observations were dropped from analysis.
